# Epidemiology of Musculoskeletal Injuries in the Navy: A Systematic Review

**DOI:** 10.3389/ijph.2022.1605435

**Published:** 2022-12-01

**Authors:** Tian-Tian Chang, Qi-Hao Yang, Pei-Jie Chen, Xue-Qiang Wang

**Affiliations:** ^1^ Department of Sport Rehabilitation, Shanghai University of Sport, Shanghai, China; ^2^ Department of Rehabilitation Medicine, Shanghai Shangti Orthopaedic Hospital, Shanghai, China; ^3^ Shanghai Key Lab of Human Performance, Shanghai University of Sport, Shanghai, China

**Keywords:** incidence, prevalence, epidemiology, musculoskeletal injuries, navy

## Abstract

**Objectives:** This study aimed to critically review the results of recent studies that investigated the epidemiology of noncombat-related musculoskeletal injuries (MSIs) in the Navy.

**Methods:** A systematic search was conducted of three major databases (Pubmed, Embase, and Cochrane) to identify epidemiological studies on MSIs in the Navy. Study selection and risk of bias assessment were conducted.

**Results:** The overall prevalence of MSIs ranged from 12.69% to 48.81%. And the prevalence of head and face injuries, upper extremity injuries, spine injuries, chest injuries, and lower extremity injuries were 0.11%–0.66%, 0.53%–11.47%, 0.75%–12.09%, 0.43%–0.95%, and 0.4%–21.17%, respectively. For the specific MSIs, the incidence ranged from 0.03/1000 person-years to 32.3/1000 person-years in the Navy and Marines. The ankle-foot, lumbopelvic, knee and lower leg, and shoulder were identified as the most frequent location for MSIs.

**Conclusion:** This systematic review summarized that the Navy population had a high prevalence of MSIs. And different risk factors for MSIs varied from different anatomic locations. This systematic review also provided valuable information on MSIs for sports medicine specialists.

## Introduction

Musculoskeletal injuries (MSIs) refer to muscular or skeletal system injuries, characterized primarily by pain, discomfort, and limited mobility [1, 2]. The physical fitness of military personnel faces high-intensity tests in daily training and combat environments that increase their risk of MSIs [3]. It has become the most important disease affecting non-combat attrition in the army and also brings a heavy economic burden [4, 5]. Besides, MSIs cause functional impairment, disability, and depletion of the military population [6, 7]. About 55% of veterans were diagnosed with one or more musculoskeletal disorders and related pain between 2001 and 2011, according to a survey by the US Department of Veterans Services [8]. MSIs are also a major health problem among active-duty military personnel, with an estimated 1.6 million injury-related medical visits per year, the second leading cause of medical visits [9–12].

The Navy faces great challenges due to the harsh external environment while serving at sea or performing ocean-going missions. The high incidence of MSIs among Naval officers and soldiers is related to their special military training and living environment [13, 14]. The environments of high temperature, high humidity, high salt, high noise, high radiation, and high concentration of harmful gas during the long sea voyage have a significant impact on the occurrence of training injuries among officers and soldiers [15, 16]. In the United States Marine Corps, MSIs occur primarily in young Marines who require strenuous exercise during training [17]. Therefore, for medical and financial personnel in the Navy population, effective and accurate injury incidence is necessary to illustrate the periodicity and widespread condition of injuries among the Navy’s diverse populations [18]. Such studies are used to evaluate and determine the need for health attention. By understanding the incidence, location, and cause of MSIs in the Navy, the proper and effective treatment of noncombat-related MSIs in daily training will greatly maintain the Navy’s combat effectiveness. Currently, although some studies have investigated the sources of MSIs in the Navy and injuries types, the lack of consistency in the epidemiology of MSIs across studies suggests the need for a comprehensive evaluation of the current status of Naval MSI epidemiology studies. To the authors’ knowledge, there is no review of the epidemiology of MSIs at various sites in the Navy population. If common MSI sites can be identified, attention can be focused on potential ways to indirectly address the factors that cause injury at these sites, thereby mitigating their impact and reducing the risk of MSIs in the Navy. Therefore, the primary purpose of this study is to identify, critically review, and synthesize the results of recent studies that have investigated the epidemiology of noncombat-related MSIs in the Navy.

## Methods

### Search Strategy

The primary search was systematically performed using three electronic databases (Pubmed, Embase, and Cochrane) to obtain research on the epidemiology of MSIs in the Navy. All databases were searched from inception to 13 April 2022. The key search phrase for identifying potentially relevant articles was related to Navy, Naval, coast guard, submarine, sailor, seaman, Marines, injury, or injuries (Supplementary Material). In addition, the potentially relevant literature (e.g., the reference lists of included studies) was hand-searched. This systematic review protocol was registered in Open Science Framework and can be accessed at: https://osf.io/d2g3h.

### Study Selection

**FIGURE 1:**
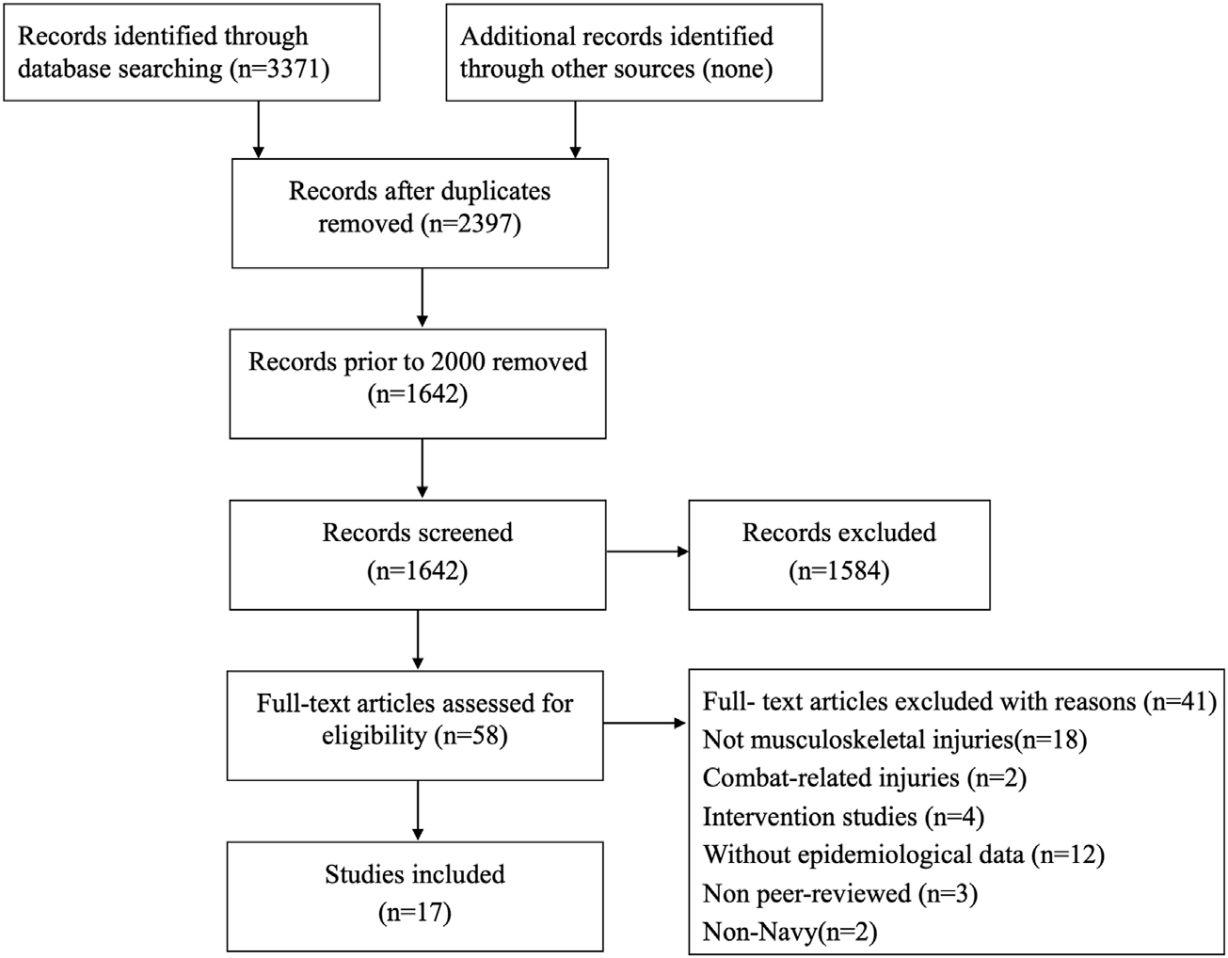
Flowchart of systematic review (worldwide, 2000–2021). Note: The flow diagram was based on the PRISMA [62].

The selection/inclusion process is shown in a flowchart in Figure 1. Only literature written in English was considered eligible for inclusion. This systematic review included original articles that reported the incidence or prevalence of MSIs among the Navy or Marines or that provided sufficient data to allow the calculation of injury incidence or prevalence. Studies meeting any of the following criteria were excluded: 1) they were published as a conference abstract, dissertations, or book chapters; 2) studies were case reports, editorials, and intervention studies; 3) studies that reported the incidence or prevalence of MSIs during war actions or natural and manmade disasters; and 4) studies that did not contain raw data (e.g., person-years and follow-up duration) or could not be extracted from tables and figures.

Two reviewers independently reviewed the study titles and abstracts that were initially identified based on the inclusion criteria. When the titles and abstracts met the inclusion criteria, the full text of the article was reviewed. Full articles were also read when articles could not be excluded based on the title and the abstract. Any discrepancies between the review authors regarding the selection process were resolved by discussion or a third external reviewer.

### Data Sources and Searches

The two reviewers independently extracted the information of the included studies, including the study type, country of origin, subject characteristics (e.g., the surveyed population, gender, age, recruitment time, and analyzed number of samples), epidemiological data (e.g., incidence rate, prevalence rate, number of the injuries, anatomic location of the injuries, injury type, and risk factors), and assessment method for the epidemiological data. In this systematic review, the prevalence was calculated as the proportion of participants with MSIs. The incidence rate of MSIs was expressed as the number of injury cases per 1000 person-years. If the study did not directly report the person-years, the number of person-years was obtained using the analyzed number of samples and the follow-up time, thereby calculating the incidence rate. The prevalence of MSIs was reported by dividing the number of injury cases by the total sample size.

### Risk of Bias Assessment

The National Institutes of Health Quality Assessment Tool for Observational Cohort and Cross-sectional studies was used to assess the quality of all included articles by two review authors [19]. Similarly, any disagreement between the review authors was resolved by discussion or a third reviewer to reach a consensus for all quality appraisals. This quality assessment tool included 14 items, with each item focusing on the key concepts of internal validity. The assessment was based on whether the included studies conformed to the 14 criteria. This tool has been previously shown to estimate the risk of potential for selection bias, information bias, measurement bias, and confounding well. The total score for each study was interpreted as good (scores 10‒14), fair (scores 6‒9), or poor (scores 0‒5).

## Results

### Descriptive Characteristics of the Studies

As shown in Figure 1, the literature search yielded 3371 articles, of which 974 duplicates were excluded. A total of 755 studies published before 2000 were excluded to ensure that the data reflected the most recent information on the epidemiology of MSIs in the Navy. Subsequently, 1584 studies were excluded by reading the title and abstract. The full texts of 58 studies were evaluated for eligibility, of which 17 studies met the inclusion criteria and 41 studies were excluded. After full-text reading, the main reasons for exclusion were as follows: war-related MSIs, disaster-related MSIs, not reporting the incidence or prevalence of MSIs or insufficient data.

A total of 17 studies (published between 2000 and 2021, the epidemiology data were collected between 1980 and 2016) were included in this systematic review. In terms of the study design, 1 study [20] was a prospective study, 9 studies [21–29] used a retrospective study design, and 7 studies [13, 30–35] were cross-sectional studies. Fifteen articles originated from the United States, and the 2 other studies [13, 34] originated from China and Brazil. Of those, 9 articles [13, 20, 21, 28, 30–34] reported the sample size ranging from 210 to 424596, and 5 studies [13, 30, 32–34] reported participants’ age; the mean age ranged from 19.32 to 28.80 years. Five studies [13, 20, 21, 28, 30] involved both female and male participants, with the percentage of females varying between 11.77% and 39.73%, but 3 studies [32–34] recruited only male subjects. Only 1 study [13] used a questionnaire to collect prevalence or incidence data, whereas 16 studies used medical charts or medical epidemiology databases or physical examination. A total of 3 studies [20, 28, 36] recruited Navy cadets or midshipmen, 12 studies included Navy and/or Marines crewmembers, and 2 studies [32, 34] recruited Naval active-duty members and students or recruits (Table 1).

**TABLE 1:** Characteristics of included studies (worldwide, 2000–2021).

Author, year	Study design	Recruited Time	Country	Participant	Sample size (Male, Female)	Age	Number of injury regions	Method for evaluating prevalence or incidence of injuries
Mullinax et al., 2021	Retrospective	2009–2015	United States	Navy and Marines	424596(NR)	NR	1(back pain)	Military health system data repository
Fraser et al., 2021	Retrospective	2005–2006	United States	Navy and Marines	NR	NR	1(lateral ankle sprain)	Defense medical epidemiology database
Lovalekar et al., 2020	Cross- sectional	2014.7–2015.7	United States	Marines	302(218M, 84F)	M:22.4 ± 2.6, F: 22.6 ± 2.8	10(head/face; shoulder; wrist; hand and fingers; thoracic; lumbopelvic; hip; thigh; knee; lower leg; ankle; foot and toes)	Certified athletic trainers and navy corpsmen diagnosis
Hiebert et al., 2020	Cross- sectional	2015–2016	United States	Navy and Marines	12000	NR	8(shoulder; arm or hand; cervical; Thoracic; Lumbopelvic; hip; knee; ankle)	Medical charts
Gun et al. 2018	Retrospective	2006-2015	United States	Navy and Marines	NR	NR	1(sternoclavicular joint dislocation)	Defense medical epidemiology database
Lovalekar et al., 2017	Cross- sectional	2008–2016	United States	Naval special warfare personnel (including SEAL operators, SQT students, SWCC operators, and crewman CQT students	920 (920M,0F)	SEAL:28.8±6, SQT: 24.0±2.8, SWCC: 27.2±5.2, CQT: 22.8±3.1	17(head/face; shoulder; upper arm; elbow; forearm; wrist; hand and fingers; chest; cervical; thoracic; lumbopelvic; hip; thigh; knee; lower leg; ankle; foot and toes	Medical charts
Lopes et al. 2017	Cross- sectional	2016.1–2016.3	Brazil	Naval Academy cadets	545(394M,151F)	21±2	7(low back; hip; Thigh; Knee; Lower leg; ankle; foot)	Questionnaire
Lovalekar et al. 2016	Cross- sectional	2008–2013	United States	Naval special warfare sea, air and land operators	210(210M,0F)	28.1±6	14(shoulder; upper arm; forearm; wrist; hand and fingers; chest; cervical; thoracic; lumbopelvic; thigh; knee; lower leg; ankle; foot and toes)	Medical charts
Qi et al., 2016	Cross-sectional	2014.5–2014.7	China	Navy(including active-duty crewmembers, marines and recruits)	6769(6769M,0F)	crewmembers: 20.88±2.96; marines:20.2±2.19 recruits:19.32±1.78	7(head/face; upper and lower arm, hand and wrist; spine and back; thigh; knee and lower leg; ankle and foot)	Medical records
Showery et al., 2016	Retrospective	2005–2014	United States	Navy	NR	NR	1(knee osteoarthritis)	Defense medical epidemiology database
Hsiao et al., 2015	Retrospective	1999–2008	United States	Navy and Marines	NR	NR	1(shoulder impingement)	Defense medical epidemiology database
Schoenfeld et al. 2013	Retrospective	2001–2010	United States	Navy and Marines	NR	NR	1(lumbar spine fractures)	Defense medical epidemiology database
Hsiao et al. ,2012	Retrospective	1999–2008	United States	Navy and Marines	NR	NR	1(clavicle fractures)	Defense medical epidemiology database
Litow et al., 2007	Cross- sectional	2005–2006	United States	Navy and Marines	NR	NR	NR	Medical Board Online Tri-Service Tracking 2006
Boling et al., 2010	Prospective	2005–2008	United States	Naval Academy cadets	1525(919M, 606F)	NR	1(patellofemoral pain syndrome)	Medical charts
Gwinn et al., 2000	Retrospective	1991–1997	United States	Naval academy midshipmen	24501(21617M, 2884F)	NR	1(anterior cruciate ligament injury)	Medical charts
Smith et al., 2000	Retrospective	1980–1992	United States	Navy and Marines	NR	NR	NR	Navy Medical Information Management Center

Note: F, Female; M, Male; NR, not reported; SEAL operators, Sea, Air, and Land operators; SQT students, SEAL Qualification Training students; SWCC operators, Special Warfare Combatant-craft Crewman operators; CQT students, Crewman Qualification Training students.

### Prevalence of Musculoskeletal Injuries

Table 2 provides a summary of the prevalence and incidence of MSIs. Seven studies [13, 21, 30–34] reported the prevalence of multi-site MSIs. The overall prevalence of MSIs ranged from 12.69% to 48.81%. Specifically, the prevalence of head and face injuries varied from 0.11 to 0.66% according to the data from 3 studies [30, 32, 34]. Five studies [30–34] showed that the prevalence of upper extremity injuries ranged from 0.53% to 11.47% (shoulder, 0.41%–7.14%; upper arm, 0.12%–0.95%; elbow, 0.43%; forearm, 0.22%–1.43%; wrist, 0.48%–1.06%; and hand and fingers, 1.43%–1.66%) and the prevalence of lower extremity injuries ranged from 0.4% to 21.17% (hip, 0.04%–3.97%; thigh, 0.87%–2.38%; knee, 0.25%–13.5%; lower leg, 1.32%–2.17%; ankle, 0.11%–4.3%; and foot, 1.43%–7.28%). In addition, 6 studies [13, 21, 30–33] reported that the prevalence of spine injuries ranged from 0.75% to 12.09% (cervical, 0.02%–1.43%; thoracic and lumbopelvic, 0.73%–12.09%). However, only 2 studies [32, 33] suggested a prevalence of 0.43%–0.95% for chest injuries.

**TABLE 2:** The incidence and prevalence rate of musculoskeletal injuries for the navy (worldwide, 2000-2021).

Autho r, year	Sam ple size	Head AND face	Shoul der	Upper arm	Elbow	Forear m	Wrist	Hand and fingers	Clavic le	Chest	Cervic al:	Thora cic	Lumbo pelvic	Hip	Thigh	Knee	Lower leg	Ankle	Foot
Mullin ax etal. 2021	424596	NR										51337(12.09)/		NR					
Fraser et al. 2021	NR	NR																/21.3 (N) 32.3(M)	NR
Lovale kar et al.2020	302	2 (0.66)/	7(2.32)	NR			2 (0.66)/	5 (1.66)/	NR			3 (0.99)/	15 (4.97)/	12 (3.97)/	3 (0.99)/	10 (3.31)/	4 (1.32)/	13 (4.3)/	22 (7.28)/
Hieber et al. 2020	12000	NR	49 (0.41)/	14(0.12)/					NR		2 (0.02)/	14 (0.17)/	NR	5 (0.04)/	NR	30 (0.25)/	NR	13 (0.11)/	NR
Gun, B et al. 2018	NR	NR							/0.03(N:0.01 9; M:0.036)	NR									
Lovale kar et al.2017	920	1 (0.11)/	35 (3.80)/	8 (0.87)/	4 (0.43)/	2 (0.22)/	6 (0.65)/	15 (1.63)/	NR	4 (0.43)/	8 (0.87)/	12 (1.30)/	34(3.7)/	16 (1.74)/	8 (0.87)/	34 (3.70)/	20 (2.17)/	33 (3.59)/	23 (2.50)/
Lopes et al. 2017	545	NR											96(17.61)/	10(1.84) /	26(4.77)/	116(22.28)/	92(16.88)/	55(10.09)/	49(8.99)/
Lovale kar et al.2016	210	NR	15 (7.14)/	2 (0.95)/	NR	3 (1.43)/	1 (0.48)/	3 (1.43)/	NR	2 (0.95)/	3 (1.43)/	4 (1.90)/	8 (3.81)/	NR	5 (2.38)/	5 (2.38)/	3 (1.43)/	6 (2.86)/	3 (1.43)/
Qi et al. 2016	6769	39 (0.58)/	NR	51(0.75)/			72(1.06)/		NR	NR	85(1.26)/			NR	63 (0.93)/	257(3.80)/		292(4.31)/	
Showe ry et al. 2016	NR	NR														/1.1 (N:1.1 6;M:0.7 0)	NR		
Hsiao, M.S., et al. 2015	NR	NR	/6.20 (N:6.0 8; M:6.44)	NR														10.4	NR
Schoen feld et al.2013	NR	NR											/0.35(N:0.29;M:0.4 6)	NR					
Hsiao, M.S., et al. 2012	NR	NR							/0.98(N:0.79;M:1.3 6)	NR								10.4	NR
Boling et al. 2010	1525	NR														206(13.50)/22	NR		
Litow et al. 2007	NR	NR	/7.78 (not reported injury location)																
Gwinn et al. 2000	24501	NR														159 (0.65)/	NR		
Smith et al. 2000	NR	NR	/9.7(not reported injury location)																

Note: the number of MSIs (prevalence, %) /incidence (1000 person-years); N, Naval; M, Marines.

### Incidence of Musculoskeletal Injuries

Eight studies explored the incidence of specific MSIs [e.g., sternoclavicular joint (SCJ) dislocation, clavicle fractures, shoulder impingement, lumbar spine fracture, patellofemoral pain syndrome (PFPS), anterior cruciate ligament (ACL) injury, knee osteoarthritis (KOA), and lateral ankle sprain (LAS)] [20, 23–28, 22]. Besides, 2 studies reported the overall incidence of MSIs for unspecified sites [29, 35]. For the specific MSIs, 2 studies [23, 27] investigated the incidence of clavicle-related injuries and reported an incidence of 0.98 per 1000 person-years for clavicle fractures and an incidence of 0.03 per 1000 person-years for SCJ dislocation. The incidence rates of shoulder impingement [25], lumbar spine fracture [26], and KOA [24] were 6.20 (Navy, 6.08; Marines 6.44), 0.35 (Navy, 0.29; Marines 0.46), and 1.10 (Navy, 1.16; Marines, 0.70)/1000 person-years for the Navy and Marines, respectively. The incidence of PFPS among Naval academy students was 22/1000 person-years [20]. The incidence of LAS was 21.3 and 32.3/1000 person-years in the Navy and Marines, respectively [22]. For non-site-specific MSIs, 2 studies reported that the overall incidence ranged from 7.78/1000 person-years to 9.7/1000 person-years [29, 35].

### Injury Location of Musculoskeletal Injuries

For Marines, Lovalekar et al. 2020 reported that 35%, 15%, 12%, and 10% of MSIs were injuries to the ankle-foot, lumbopelvic, hip, and knee, respectively [30]. The ankle-foot (30.0%) was the most common location of MSIs among the Naval Special Warfare Operators and students, followed by the shoulder (13.1%), knee (12.7%), and lumbopelvic (12.7%) [32]. Among the Naval Academy cadets, the most frequent injury locations for MSIs were ankle-foot (19%), lumbopelvic (18%), and lower leg (17%) [13]. The most prevalent regions were shoulder (23.8%), ankle-foot (14.29%), and lumbopelvic (12.7%) among the Naval Special Warfare Operators [33]. Qi et al. (2016) reported that MSIs most commonly occurred in the ankle-foot (34%), lower leg and knee (29.9%), and spine and back (9.9%) in the Navy [34].

### Injury Type of Musculoskeletal Injuries

Four studies reported the most common type of MSIs [30, 32–34]. For Marines, the most frequent injury type was pain/spasm/ache [30]. Similarly, for Naval special warfare Sea, Air, and Land Operators, the most common injury types were strain and pain/spasm/ache [33]. The most common injury types varied across the different Naval Special Warfare (NSW) personnel. The most common type of MSIs was tendinopathy/tenosynovitis/tendonitis and fracture among the NSW students and pain/spasm/ache among NSW operators [32]. And the three leading injury types among Navy and Marines were sprain, contusion, and overuse [34].

### Risk Factors for Musculoskeletal Injuries

In addition to the incidence of specific MSIs, 3 studies reported on the risk factor for specific MSIs (back pain, PFP, ACL injury) in the Naval and Marines and found that females were more likely to develop musculoskeletal disorders than males [20, 21, 28]. 6 studies investigated risk factors for specific MSIs (e.g., LAS, SCJ dislocation, clavicle fractures, KOA, shoulder impingement, lumbar spine fracture) in military personnel [22–27]. Among them, 5 studies explored the associations between gender, age, race, enlisted rank, the branch of service, and risk of developing specific MSIs [24–27]. And they all reported that Marines had a higher risk of specific MSIs than the Navy. Whether gender, age, race, and enlisted rank were risk factors for MSIs varied across diseases (see Table 1 for full details).

### Quality Appraisal of Literature

The bias risk assessment in the included studies is presented in Table 3. The quality was considered good for 5 studies and fair for 12 studies [21, 24–26, 29]. Most of the included studies met some important criteria (such as a clear research question and valid evaluation of important variables), whereas few studies achieved other criteria (such as the assessor blinding, sample size justification, and statistical power estimation). Nevertheless, most of the included studies in this systematic review were of moderate quality.

**TABLE 3:** Risk of bias for each study (worldwide, 2000–2021).

Study	Research question stated	Study populati on defined	Particip ation rate at least 50%	In and exclusion criteria reported	Sample size justificat ion or power analyses or effect sizes provided	Exposure measured prior to outcome	Time frame sufficient for expected association	Different levels of exposure examined	Exposure clearly defined, reliable and valid	Exposur e assessed more than once	Outcome clearly defined, reliable and valid	Outcome assessors blinded to exposure status	Loss to follow- up 20% or less	confoun ding variables measured and statistica l adjusted
Mullina x et al., 2021	Y	Y	Y	Y	Y	Y	Y	Y	Y	Y	Y	Y	N	Y
Fraser J. J. et al., 2021	Y	Y	Y	N	N	Y	Y	N	Y	N	Y	N	N	Y
Lovalek ar et al., 2020	Y	Y	N	Y	N	N	N	N	Y	Y	Y	Y	N	N
Hiebert R., et al., 2020	Y	Y	Y	Y	N	N	N	N	Y	N	Y	Y	N	N
Gun B. et al., 2018	Y	Y	Y	N	N	Y	Y	Y	Y	N	Y	N	N	N
Lovalek ar et al., 2017	Y	Y	Y	Y	N	N	N	N	Y	N	Y	Y	N	N
Lopes etal., 2017	Y	Y	Y	Y	N	N	N	Y	Y	Y	N	N	N	N
Lovalek ar et al., 2016	Y	Y	N	Y	N	N	N	N	Y	N	Y	Y	N	N
Qi et al. ,2016	Y	Y	Y	Y	N	N	N	Y	Y	N	Y	Y	N	N
showery et al., 2016	Y	Y	Y	Y	N	Y	Y	Y	Y	Y	Y	Y	N	Y
Hsiao M. S. et al., 2015	Y	Y	Y	N	Y	Y	Y	Y	Y	N	Y	N	N	Y
Schoenf eld et al., 2013	Y	Y	Y	Y	N	Y	Y	Y	Y	Y	Y	Y	N	Y
Hsiao M. S. et al., 2012	Y	Y	Y	N	Y	Y	Y	Y	Y	N	Y	N	N	Y
Boling M. et al.,2010	Y	Y	N	Y	N	Y	Y	N	Y	Y	Y	N	N	Y
Gwinn D. E. et al., 2000	Y	Y	Y	Y	N	Y	Y	N	Y	N	Y	N	N	N
Litow et al., 2007	Y	Y	Y	Y	N	N	N	Y	N	N	Y	Y	N	N
Smith et al., 2000	Y	Y	Y	Y	N	Y	Y	Y	Y	N	Y	Y	N	N

Note: Y, Yes; N, No or not reported.

## Discussion

This systematic review, for the first time, summarizes the evidence on the epidemiology of MSIs in the Navy. The results of this systematic review suggested that the overall prevalence of MSIs ranged from 12.69% to 48.81%. For the MSI prevalence by anatomic location, the prevalence rates of head and face injuries, upper extremity injuries, chest injuries, spine injuries, and lower extremity injuries were 0.11%–0.66%, 0.53%–11.47%, 0.43%–0.95%, 0.75%–12.09%, and 0.4%–21.17%, respectively. The overall incidence of non-site-specific MSIs ranged from 7.78/1000 person-years to 9.7/1000 person-years. For the specific MSIs, the incidence varied from 0.29/1000 person-years to 32.3/1000 person-years.

### Upper Extremity Injuries

The results of this systematic review found that the incidence of upper extremity MSIs in the Navy population could be subdivided into the shoulder, upper arm, elbow, forearm, wrist, and finger, indicating that upper limb injuries have a certain proportion in the Navy population. In a long-term epidemiological survey of Naval Special Warfare Sea, Air, and Land Operators, upper extremity MSIs were found to be the most common, and the shoulder was the site with a high incidence of upper extremity injuries [37]. Lifting was determined to be the most likely cause of upper extremity injuries. Another study focusing on the MSI incidence of Naval Special Warfare found that a significant number of upper extremity injuries are potentially preventable [32]. Qi et al. [34] analyzed the incidence of MSIs among Chinese Naval personnel in overseas military operations undergoing different training over 12 weeks and found that shipboard training is more likely to cause upper extremity injuries than combat training, which is more likely to cause fractures. Besides, the incidence of upper extremity MSIs was higher in crewmembers receiving shipboard tactical training than in recruits. The study found that blacks younger than 20 and whites older than 40 in the Marine Corps are more likely to be diagnosed with upper limb injuries; moreover, lower ranks of the female are an important risk factor for upper limb injuries [38].

Several studies have also focused on specific conditions for upper extremity MSIs, including clavicle fractures and shoulder impingement. The incidence of clavicle fracture has increased significantly in recent years, which suggests that a larger population epidemiological investigation is necessary to determine whether the incidence of certain injuries has really increased and further examine the causes and mechanisms of the increase in the number of injuries [27]. In addition to shoulder injuries caused by the cramped cabin space, wet ground, and full-body vibration conditions on ships, age played a significant role in MSIs of the upper limbs of the Navy, and age over 40 years was also an important risk factor for shoulder impingement in the Navy [25]. Another study found a greater risk of shoulder subluxation in the Navy and Marine Corps than in the Air Force, and white service members have a higher risk of posterior subluxation than other races among a population of 3,868,007 person-years [39]. Further examination of the links between occupational risk factors and different branches within the Navy is warranted.

### Torso and Clavicle-Related Injuries

The torso injuries reported in this study included chest, spine, and back. A 12-month survey of the prevalence of MSIs in the Navy cadets found that the low back is the most common site of MSIs, and women are more prone to injury than men [13]. Studies have found that women have on average 12 kg less skeletal muscle than men, and this disparity in muscle strength is further magnified during high-intensity military training [40]. Epidemiological studies have shown that living in a ship environment prone to whole-body vibration is a risk factor for musculoskeletal symptoms of low back [41, 42]. Service in the Army, Navy or Air Force was also found to be a risk factor for low back MSIs [5]. A longitudinal study covering the US Navy and Marine Corps from 2009 to 2015 found that lifestyle factors such as being overweight and smoking were associated with an increased prevalence of low-back MSIs in the US Navy and Marine Corps [21]. It was consistent with a retrospective cohort study that found soldiers who smoked and were overweight increased their chances of prolonged and recurrent back pain [43]. And this study also found a strong link between teens and smoking. Considering the high proportion of adolescents in the active Navy, this effect may lead to smoking being an important factor influencing the incidence of low-back MSIs, which will have a more adverse impact on Naval effectiveness and the efficiency of medical expenditures. Height over 1.86 m and lower extremity injuries were also significant predictors of back pain in Marines, both of which were associated with increased burden on the spine [44]. Tall Marines can increase the leverage and burden on the spine when handling heavy equipment and materials. The loss of part of the strength and proprioceptive function caused by lower extremity injury will be compensated by the spine and increase its burden, eventually causing musculoskeletal symptoms in the back [45]. Thus, ship environment, female, overweight, smoking, tall body height, and lower extremity injuries were associated with a greater odds of occurrence of low back injuries. These findings can provide information for Navy officers to develop medical care policies.

In addition to torso injuries, some studies reported the epidemiology of MSIs in the SCJ. The Navy had the lowest incidence of SCJ dislocation compared with the Army and Air Force. Males and military rank were associated with SCJ dislocations, whereas age and race were not potential risk factors [23]. Junior soldiers may encounter more rigorous training than their superiors, leading to a greater chance of dislocation. Understanding the common risk factors for SCJ dislocation can encourage the most vulnerable patients to recognize and prevent these injuries.

### Lower Extremity Injuries

The lower extremity injuries in this systematic review included hip, thigh, knee, lower leg, ankle, and foot injuries. Most MSIs were located in the lower extremity of the Navy and Marines recruits [30, 46]. The injury patterns may differ among the Naval Special Warfare personnel. Lovalakar et al. [32] observed that lower extremity MSIs were more common than upper extremity MSIs among the Sea, Air, and Land Qualification Training students, and Crewman Qualification Training students. However, the opposite was found among the Sea, Air, and Land Operators. A possible explanation may be the different training types [32]. Specifically, students were more participated in high-intensity running, while operators placed more emphasis on strength training, especially lifting. Many studies also reported a difference in injury patterns in lower extremity injuries between females and males. A higher prevalence of MSIs was found in female than in male Navy cadets [13] and Marines [30], especially in the knee, hip, and lower back. Many biomechanical and anatomical factors can contribute to gender differences in lower extremity injuries, such as the larger Q angles [47], the weaker muscle strength in lower extremity [48], the increased hip adduction and internal rotation angles [49], the increased knee valgus angle, the decreased knee flexion angles [50], the hormonal effects [51], and other altered biomechanics in women during physical activities.

The results of a study by Lope et al. [36] found that the reduced trunk endurance (maintenance duration for the prone plank test less than 60 s), decreased flexibility (the sit and reach test with a score less than 18 cm), and a history of pain (at least two sites within the past year) are predictive factors for lower extremities injuries among Naval cadets. Some studies also described the epidemiology and risk factors of lower extremity MSIs in different military services [22, 24, 32, 34]. Showery et al. [24] reported that the incidence of KOA in the Marines is higher than that in the Navy, and they also identified the risk factors for KOA in the military population, including increasing age, black race, senior military rank, and Army, Marines, and Air Force services. Fraser et al. [22] performed a retrospective cohort study to explore the risk factors for lateral ankle sprain. They concluded that female sex and military occupations are significant risk factors for LAS [22]. Among them, aviation officers are at lower risk, whereas engineers, maintenance, administration, operations/intelligence, and logistics officers are at higher risk compared with ground/Naval gunfire officers [22]. For the lower extremity injury prevention strategy, the use of foot orthoses has been shown to have a potential protective effect on Naval recruits during initial defense training [52]. Additionally, an improvement in physical fitness can decrease the rate of MSIs in the Navy [17].

### Non-Site-Specific Musculoskeletal Injuries

In addition to the specific sites of MSIs, some studies reported the epidemiology of MSIs without the reported injury location. According to the Navy Physical Evaluation Board data, MSIs are the most common form of injury, with the incidence increasing from 4.31 per 1000 person-year in 2000 to 7.78 per 1000 person-year in 2005 [35]. Peterson et al. [14] reported that the overall injury rate was 0.9–3.2 injuries per 100 personnel per month in the SEAL operators and SEAL support. China Navy crewmembers deployed at sea had higher rates of MSIs than those in port, but this was in contrast to the findings of a study that focused on the incidence of MSIs in the US Navy [53]. Tactical training-related injuries were not included in the study of Chinese Navy crew members, and the military training tasks assigned by the two armies were also quite different. The prevalence of MSIs at the Naval Academy increased with the academic year, especially in the first to second year [13]. The most injury-inducing scenario was when participants were engaged in daily high-intensity training, followed by recreational physical activity. These findings indicated the need to assess the protocols and self-training programs followed by active personnel, as well as to improve awareness and strategies for avoiding MSIs.

Regarding gender differences, many studies demonstrated that females are more likely to suffer from MSIs than males in the general population in sports activities [54, 55]. Similar to gender differences in the prevalence of MSIs among the Navy, Bell et al. [56] and Jones et al. [57] also reported that females have a higher risk of MSIs (relative risk: 2.1) than males in the Army. As mentioned above, these findings suggested that females are more likely to have MSIs than males in the military population. Therefore, modifiable gender-specific characteristics must be identified to develop corresponding interventions to reduce the risk of MSIs in females. In addition to some biomechanical and anatomical factors associated with MSIs, previous research suggested that psychosocial parameters (e.g., gender socialization) may contribute to the gender difference in the incidence and prevalence of MSIs [58]. However, Dos et al. [59] performed a systematic review and found that being older, overweight or obese, having previous injuries, and running performance were risk factors of MSIs in the military population but not gender, ethnicity, and smoking. Another explanation may be related to the low level of physical fitness and low physical activity level in the past [57, 60]. Bell et al. [56] also demonstrated that physical fitness is a key risk factor for MSIs rather than gender after controlling for potential confounders (including demographics, body composition, and initial physical fitness). These results suggested a customized program for improving the physical fitness of females to help reduce the MSI risk in military training. In addition, in a systematic review, Wardle et al. [61] summarized several preventive strategies for MSIs in military population and recommended improving physical fitness, decreasing the amount of physical activity volume, and increasing awareness.

### Limitation

Only three electronic databases were retrieved in this systematic review. A small number of studies were included in this systematic analysis. The quality evaluation of the literature included in this study was mostly moderate, and the number of studies with high quality was few. Given the heterogeneous nature of the included literature, no meta-analysis was conducted in this study. Finally, despite our systematic and comprehensive search, many of the included studies analyzed the data from the same epidemiology database (US defense medical epidemiology database).

### Conclusion

This first systematic review summarizes evidence on the epidemiology of MSIs in the Navy. This study showed that MSIs are common in the Navy with overall prevalence ranging from 12.69% to 48.81%. For MSI prevalence by anatomic location, the prevalence rates of head and face injuries, upper extremity injuries, spine injuries, chest injuries, and lower extremity injuries were 0.11%–0.66%, 0.53%–11.47%, 0.75%–12.09%, 0.43%–0.95%, and 0.4%–21.17%, respectively. There are different risk factors for MSIs in different anatomic locations. This systematic review can provide valuable information on MSIs for sports medicine specialists and athletic trainers. However, limited evidence is available on noncombat-related MSIs in the Navy, so more research on the prevalence and incidence of MSIs by anatomic location and specific pathologies is needed in the future.
