# Supplementary material for: Epidemiology of Musculoskeletal Injuries in the Navy: A Systematic Review
Source: Int J Public Health. 2022 Dec 1;67:1605435. doi: 10.3389/ijph.2022.1605435 (PMC9751041; doi:10.3389/ijph.2022.1605435)
Supplement: Supplementary file 1 [file DataSheet1.docx]

**1. Search Strategy for Pubmed**

#1 "injury"[MeSH Terms] OR injury[Title/Abstract] OR injuries[Title/Abstract]

#2 navy[Title/Abstract] OR naval[Title/Abstract] OR coast guard[Title/Abstract] OR submariner*[Title/Abstract] OR navy personnel[Title/Abstract] OR Personnel, Navy[Title/Abstract] OR Sailors[Title/Abstract] OR Sailor[Title/Abstract] OR Marines[Title/Abstract] OR seaman[Title/Abstract]

#3 #1 AND #2

**2.** **Search Strategy for Embase**

#1 'injury'/exp

#2 'navy'/exp

#3 naval:ti,ab,kw OR submariner*:ti,ab,kw OR 'coast guard':ti,ab,kw OR sailor*:ti,ab,kw OR marines:ti,ab,kw OR seaman:ti,ab,kw OR navy:ti,ab,kw

#4 injury:ti,ab,kw OR injuries:ti,ab,kw

#5 #1 OR #4

#6 #2 OR #3

#7 #5 AND #6

**3. Search Strategy for Cochrane Library**

#1 MeSH descriptor: [Wounds and Injuries] explode all trees

#2 navy:ti,ab,kw OR naval:ti,ab,kw OR coast guard:ti,ab,kw OR submariner*:ti,ab,kw OR navy personnel:ti,ab,kw

#3 Sailors:ti,ab,kw OR Sailor:ti,ab,kw OR Marines:ti,ab,kw OR seaman:ti,ab,kw OR Personnel, Navy:ti,ab,kw

#4 #2 OR #3

#5 injury:ti,ab,kw OR injuries:ti,ab,kw

#6 #1 OR #5

#7 #4 AND #6
